# Use of a baseline risk score to identify the risk of serious infectious events in patients with rheumatoid arthritis during certolizumab pegol treatment

**DOI:** 10.1186/s13075-017-1466-y

**Published:** 2017-12-15

**Authors:** Jeffrey R. Curtis, Kevin Winthrop, Cathy O’Brien, Matladi N. Ndlovu, Marc de Longueville, Boulos Haraoui

**Affiliations:** 1 University of Alabama at Birmingham, Birmingham, AL USA; 2 Oregon Health and Science University, Portland, OR USA; 3 UCB Pharma, Brussels, Belgium; 4 Department of Medicine, Centre Hospitalier de l’Université de Montréal, Montréal, QC Canada

**Keywords:** Certolizumab pegol, Safety, Global risk score, Comorbidity, Serious infection

## Abstract

**Background:**

The risk of serious infectious events (SIEs) is increased in patients with rheumatoid arthritis (RA). The aim of this study was to develop an age-adjusted comorbidity index (AACI) to predict, using baseline characteristics, the SIE risk in patients with RA treated with certolizumab pegol (CZP).

**Methods:**

Data of CZP-treated patients with RA were pooled from the RAPID1/RAPID2 randomized controlled trials (RCT CZP) and their open-label extensions (All CZP). Predictors of the first SIE were examined using multivariate Cox models. The AACI was developed by assigning specific weights to patient age and comorbidities on the basis of relative SIE risk. SIE rates were predicted using AACI score and baseline glucocorticoid use, and they were compared with observed rates. The percentage of patients in each SIE risk group achieving low disease activity (LDA)/remission was examined at 1 year of treatment.

**Results:**

Among 1224 RCT CZP patients, 40 reported ≥ 1 SIE (incidence rate [IR] 5.09/100 patient-years [PY]), and 201 of 1506 All CZP patients reported ≥ 1 SIE (IR 3.66/100 PY). Age ≥ 70 years, diabetes mellitus, and chronic obstructive pulmonary disease/asthma made the greatest contributions to AACI score. SIE rates predicted using AACI and glucocorticoid use at baseline showed good agreement with observed SIE rates across low-risk and high-risk groups. At 1 year, more high-risk All CZP patients than low-risk All CZP patients reported SIEs (IR 8.4/100 PY vs. IR 3.4/100 PY). Rates of LDA/remission were similar between groups.

**Conclusions:**

AACI and glucocorticoid use were strong baseline predictors of SIE risk in CZP-treated patients with RA. Predicted SIE risk was not associated with patients’ likelihood of clinical response. This SIE risk score may provide a valuable tool for clinicians when considering the risk of infection in individual patients with RA.

**Trial registration:**

ClinicalTrials.gov, NCT00152386 (registered 7 September 2005); NCT00160602 (registered 8 September 2005); NCT00175877 (registered 9 September 2005); and NCT00160641 (registered 8 September 2005).

**Electronic supplementary material:**

The online version of this article (doi:10.1186/s13075-017-1466-y) contains supplementary material, which is available to authorized users.

## Background

Anti-tumor necrosis factor (anti-TNF) agents are often the first class of biologic drugs prescribed to patients with rheumatoid arthritis (RA), providing an effective therapeutic option that improves clinical, radiographic, and functional outcomes [1]. Owing to their immunomodulatory action, anti-TNFs have been linked to an increased risk of serious infectious events (SIEs), although the strength of this association remains a topic of debate [2–5]. In light of these concerns, biologic registries have been established in several countries to examine the long-term safety of anti-TNFs and to investigate how different patient characteristics affect the risk of serious infection [6–9].

In addition, older age and specific comorbidities that are relatively prevalent in the RA population, such as diabetes mellitus or chronic obstructive pulmonary disease (COPD), have been associated with an increased risk of SIEs during anti-TNF therapy [9–14]. Concomitant treatment with systemic glucocorticoids has also been linked to an increased susceptibility to infection [6, 8, 12, 15]. In clinical practice, physicians have to balance the benefits of the different available treatments against their overall risk, as well as the risks associated with the characteristics of each patient. This often involves the complex task of extrapolating data from study populations to the individual patient level. Infection risk scores, which summarize the relative contributions of various patient characteristics in a single composite measure, may help clinicians to anticipate the potential risk of SIEs in individual patients and make better-informed treatment decisions when balancing the expected therapeutic risks and benefits for an individual patient [11, 16, 17].

Certolizumab pegol (CZP) is a PEGylated, Fc-free anti-TNF approved for the treatment of adult patients with moderate to severe active RA [18–21]. Using baseline patient characteristics, we developed and tested an age-adjusted comorbidity index (AACI) to predict the risk of SIEs in patients with RA at the start of CZP treatment. In addition, we investigated if the predicted SIE risk was associated with the likelihood of clinical response at 1 year of CZP treatment.

## Methods

### Patient population and study design

**Fig. 1:**
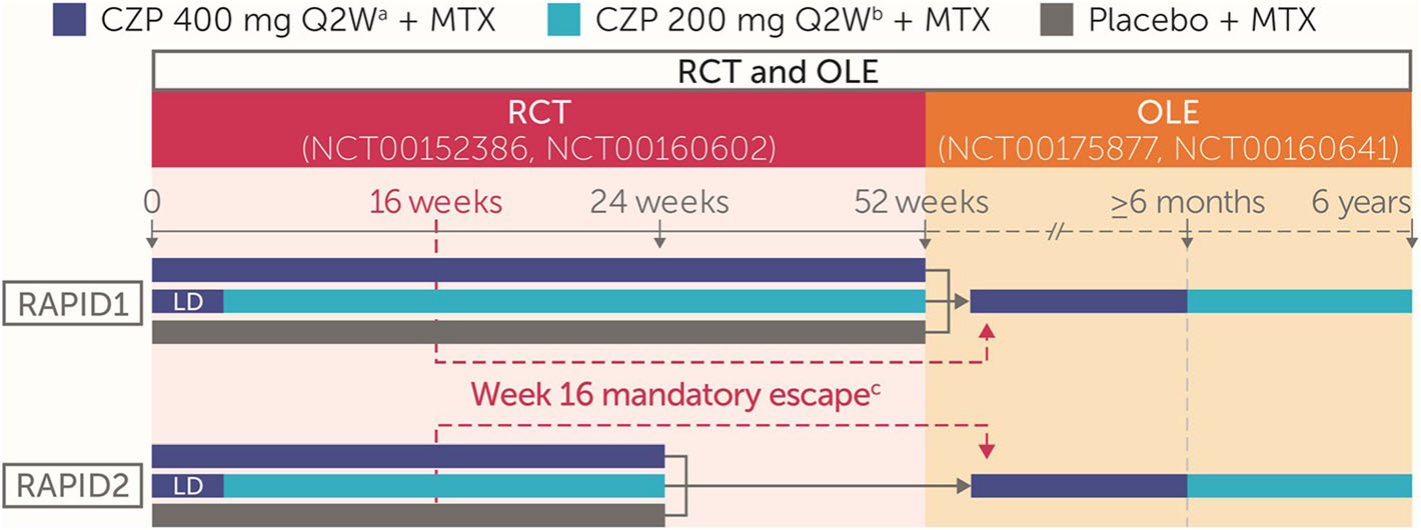
Rheumatoid Arthritis PreventIon of structural Damage 1 (RAPID1) and RAPID2 study design. Patients were randomized 2:2:1 to CZP 400 mg Q2W, CZP 200 mg Q2W, or placebo Q2W, respectively, in combination with MTX. The loading dose (LD) was CZP 400 mg at weeks 0, 2, and 4. ^a^Twice the registered CZP dose. ^b^Registered CZP dose. ^c^At week 16, American College of Rheumatology 20% improvement nonresponders at weeks 12 and 14 were withdrawn from the RCTs; these patients, as well as those who completed the RCTs, were allowed to enter the OLEs, receiving CZP 400 mg Q2W + MTX for ≥ 6 months before being switched to CZP 200 mg Q2W + MTX. *CZP* Certolizumab pegol, *Q2W* Every 2 weeks, *Q4W* Every 4 weeks, *MTX* Methotrexate, *RCT* Randomized controlled trial, *OLE* Open-label extension

To develop an AACI predictive of SIE risk in patients with RA initiating CZP treatment, we pooled baseline data from anti-TNF naive patients with RA who participated in the Rheumatoid Arthritis PreventIon of structural Damage 1 (RAPID1) and RAPID2 randomized controlled trials (RCTs; ClinicalTrials.gov, NCT00152386 and NCT00160602, respectively) and their respective open-label extensions (OLEs; NCT00175877 and NCT00160641, respectively). These pivotal registration studies have been described in detail elsewhere [20–23], and their study design is summarized in Fig. 1.

Oral glucocorticoids (≤10 mg/day prednisone equivalent) were permitted, provided that doses remained stable within 28 days of baseline and throughout the RCTs; doses were allowed to change during the OLEs. Additional details concerning patients’ eligibility criteria and permitted medications are reported elsewhere [20, 21].

### Definition of serious infectious events and patient groups analyzed

SIEs were classified according to the Medical Dictionary for Regulatory Activities (MedDRA) version 9.0. The definition of SIE encompassed the regulatory definition of serious adverse event (SAE) of infection [24] plus any medical events deemed important by the investigator, regardless of infection severity. All suspected SIEs were subsequently expert-reviewed by an external independent safety steering committee that classified SIEs by the regulatory definition of SAE of infection, with an additional criterion of the need for intravenous antibiotics [25].

For each patient, analyses included the first SIE that occurred after the first dose of CZP and up to 84 days (six times the half-life of CZP) after the last study dose or patient withdrawal; any subsequent SIEs were not included. Two overlapping patient groups were analyzed: (1) patients randomized to CZP in the RCTs (RCT CZP; only SIEs occurring during the RCTs were included) and (2) all patients treated with CZP during the RCTs and/or OLEs, including RCT placebo completers switched to CZP at the start of OLE as well as patients withdrawn from the RCTs at week 16 who reconsented to enter the OLE (All CZP; SIEs occurring during the RCTs or OLEs were included).

### Derivation of age-adjusted comorbidity index

An AACI was developed to predict the influence of baseline age and medically treated comorbidities on the risk of SIEs during CZP treatment. The AACI was conceptually similar to the Charlson comorbidity index [26], but it was designed to better reflect the age and specific comorbidity burden of an RA patient population similar to participants in the RAPID1/RAPID2 trials. Age at baseline (<50, ≥ 50 to < 60, ≥ 60 to < 70, and ≥ 70 years) and the most frequent medically treated comorbidities (diabetes mellitus, COPD/asthma, cardiac disorder [including coronary artery disease and heart failure], hypertension, hyperlipidemia, thyroid disorder, osteoporosis, and depression) were considered for inclusion in the AACI. Medically treated comorbidities were identified on the basis of patients’ medical histories or medications at baseline.

Using the RCT CZP data, the weight attributed to each baseline covariate in the AACI was derived by including all candidate covariates (described below) in a multivariate Cox proportional hazards model to estimate the relative risk of SIEs associated with each factor. The same analysis was repeated using the All CZP data. The two HRs obtained for each covariate in the RCT CZP and All CZP analyses were averaged to assign the corresponding weight (HR <1.2, weight = 0; HR ≥ 1.2 and < 1.5, weight = 1; HR ≥ 1.5 and < 2.5, weight = 2; HR ≥ 2.5 and < 3.5, weight = 3). An individual patient’s AACI score corresponded to the sum of the weights associated with their age and specific comorbidities (Table 1 and Additional file 1: Table S1).

**Table 1:** Relative serious infectious event risk associated with baseline age categories and medically treated comorbidities included in age-adjusted comorbidity index

		HR (95% CI)		Weight in the AACI^a^
Category		RCT CZP (*n* = 1224)	All CZP (*n* = 1506)	
Age, years	<50	Reference	Reference	0
	≥50 to < 60	1.29 (0.58–2.87)	1.39 (0.99–1.96)	1
	≥60 to < 70	1.14 (0.44–2.94)	1.40 (0.92–2.12)	1
	≥70	2.18 (0.70–6.84)	2.93 (1.69–5.09)	3
Diabetes mellitus		1.98 (0.59–6.58)	1.61 (0.90–2.89)	2
COPD/asthma		2.67 (0.77–9.27)	1.29 (0.56–2.97)	2
Cardiac disorder		N/C	1.33 (0.52–3.43)	0
Hypertension		1.34 (0.69–2.63)	0.96 (0.70–1.32)	0
Hyperlipidemia		2.39 (0.82–6.93)	1.47 (0.81–2.67)	2
Thyroid disorder		N/C	0.87 (0.42–1.78)	0
Osteoporosis		2.30 (0.98–5.39)	1.16 (0.73–1.86)	2
Depression		1.00 (0.22–4.51)	1.46 (0.75–2.83)	1

*Abbreviations: SIE* Serious infectious event, *AACI* Age-adjusted comorbidity index, *CZP* Certolizumab pegol, *COPD* Chronic obstructive pulmonary disease, *N/C* Not calculable (no patients with the indicated comorbidity experienced an SIE during the randomized controlled trials), *RCT CZP* Patients randomized to CZP in the RAPID1/RAPID2 randomized controlled trials, *All CZP* All patients treated with CZP during the RAPID1/RAPID2 randomized controlled trials and/or open label extensions

HRs were derived from a Cox proportional hazards model fitted with the indicated age categories and medically treated comorbidities; no other baseline covariates were included

^a^Weight of each category in the AACI was based on the average HR between the RCT CZP and All CZP populations (HR ≥ 1.2 and < 1.5, weight = 1; HR ≥ 1.5 and < 2.5, weight = 2; HR ≥ 2.5 and < 3.5, weight = 3)

### Evaluation of risk of SIEs

Observed incidence rates (IRs) of SIEs per 100 patient-years (PY) together with 95% CIs were calculated. Time at risk was measured from initiation of CZP up to the occurrence of the first SIE, or the total time at risk was measured for patients without SIEs (up to 84 days after the last study dose or patient withdrawal). Placebo-treated patients did not contribute any data to the analysis until they entered the OLE and began treatment with CZP.

To identify all clinically relevant predictors of SIEs, a stepwise Cox proportional hazards model was fitted with AACI categories (0, 1, ≥ 2) and additional baseline covariates: body mass index (BMI; < 20, 20–30, or > 30 kg/m^2^), 28-joint Disease Activity Score with C-reactive protein (DAS28[CRP]), disease duration (<2 or ≥ 2 years), Health Assessment Questionnaire Disability Index (HAQ-DI), joint erosion score (computed as log[erosion score] and included as a proxy for disease severity), methotrexate dose (≤15 or > 15 mg/week), and systemic glucocorticoid use (yes or no). Covariates were retained in the Cox model if there was an indication of a link with the occurrence of the first SIE (*p* ≤ 0.25); covariates with *p* ≤ 0.05 were identified as risk factors. All *p* values were nominal only. RCT CZP and All CZP patients were then categorized into six different risk groups based on AACI categories (0, 1, ≥ 2) and systemic glucocorticoid use (yes or no).

The Kaplan-Meier estimator was used to analyze the time to first SIE. Predicted SIE rates were based upon each patient’s covariate distribution and were obtained at regular time intervals up to the date of the last recorded SIE in RCT CZP (day 301) and up to 2 years in the All CZP group. The ability of the model to discriminate between risk groups was evaluated by calculating the c-index [27]. As a sensitivity analysis, the Rheumatoid Arthritis Observation of Biologic Therapy (the German biologics register) (RABBIT) Risk Score [17] was tested in the All CZP group (*see* Additional file 1: Methods).

### Clinical outcomes

For each of the six SIE risk groups in All CZP, the percentage of patients with low disease activity (LDA; DAS28[CRP] ≤ 2.7, Clinical Disease Activity Index [CDAI] ≤ 10, and Simplified Disease Activity Index [SDAI] ≤ 11) and in remission (DAS28[CRP] < 2.3, CDAI ≤ 2.8, and SDAI ≤ 3.3) was evaluated at 1 year of CZP exposure. Missing data were imputed using nonresponder imputation.

## Results

### Patient population and overall incidence of SIEs

The anti-TNF naive RA population pooled from RAPID1 and RAPID2 comprised 1224 patients in the RCT CZP group, with a total CZP exposure of 798.5 PY and median exposure per patient of 0.5 PY. The All CZP group comprised 1506 patients (including 282 of 311 RCT placebo patients and 1074 of 1224 RCT CZP patients who reconsented to enter the OLEs) with a total CZP exposure of 5778.6 PY and median exposure per patient of 4.8 PY. Demographic characteristics, baseline disease activity, and the prevalence of medically treated comorbidities were similar between the two patient groups (Table 2). Over 50% of patients in each group used systemic glucocorticoids at baseline.

**Table 2:** Baseline patient characteristics

		RCT CZP (*n* = 1224)	All CZP (*n* = 1506)
Female sex, *n* (%)		1008 (82.4)	1245 (82.7)
Age, years, *n* (%)	<50	470 (38.4)	580 (38.5)
	≥50 to < 60	440 (35.9)	546 (36.3)
	≥60 to < 70	247 (20.2)	300 (19.9)
	≥70	67 (5.5)	80 (5.3)
Disease duration (years), *n* (%)	<2	261 (21.3)	323 (21.4)
	≥2	963 (78.7)	1183 (78.6)
DAS28(CRP), mean (SD)		6.20 (0.83)	6.20 (0.85)
HAQ-DI, mean (SD)		1.65 (0.60)	1.65 (0.60)
CRP, mg/L, median (IQR)		15.0 (6.0–32.0)	15.0 (6.0–32.0)
Rheumatoid factor, IU/ml, median (IQR)		68.3 (18.9–201.1)	70.0 (19.4–200.0)
mTSS, median (IQR)		20.0 (5.5–54.7)	20.0 (6.0–55.0)
BMI, kg/m^2^, *n* (%)	<20	83 (6.8)	104 (6.9)
	20–30	833 (68.1)	1024 (68.0)
	>30	308 (25.2)	378 (25.1)
Glucocorticoid dose, mg/day, *n* (%)	0	518 (42.3)	637 (42.3)
	>0–5	332 (27.1)	402 (26.7)
	>5	374 (30.6)	467 (31.0)
Systemic MTX dose, mg/week, *n* (%)	≤15	1018 (83.2)	1261 (83.7)
	>15	206 (16.8)	245 (16.3)
Medically treated comorbidities, *n* (%)^a^			
Diabetes mellitus		47 (3.8)	53 (3.5)
COPD/asthma		34 (2.8)	38 (2.5)
Cardiac disorder^b^		15 (1.2)	17 (1.1)
Hypertension^c^		387 (31.6)	465 (30.9)
Hyperlipidemia		55 (4.5)	66 (4.4)
Thyroid disorder		55 (4.5)	63 (4.2)
Osteoporosis		92 (7.5)	113 (7.5)
Depression		37 (3.0)	45 (3.0)

*Abbreviations: CZP* Certolizumab pegol, *RCT* Randomized controlled trial, *DAS28(CRP)* 28-joint Disease Activity Score with C-reactive protein, *CRP* C-reactive protein, *HAQ-DI* Health Assessment Questionnaire Disability Index, *mTSS* Modified total Sharp score, *MTX* Methotrexate, *BMI* Body mass index, *COPD* Chronic obstructive pulmonary disease, *RCT CZP* Patients randomized to CZP in the RAPID1/RAPID2 randomized controlled trials, *All CZP* All patients treated with CZP during the RAPID1/RAPID2 randomized controlled trials and/or open label extensions

^a^Medically treated comorbidities were identified on the basis of patients’ medical histories and medications at baseline

^b^Cardiac disorder included coronary artery disease and heart failure

^c^Hypertension included patients with history of cerebrovascular disorder (*n* = 5 in RCT CZP and *n* = 5 in All CZP) and transient ischemic attack (*n* = 4 in RCT CZP and *n* = 5 in All CZP)

During the RCTs, 40 of 1224 RCT CZP patients reported ≥ 1 SIE (IR 5.09/100 PY [3.64–6.93]). Over the combined RCT and OLE periods, 201 of 1506 All CZP patients reported ≥ 1 SIE (IR 3.66/100 PY [3.17–4.21]). Pneumonia and cellulitis were the most common SIEs (39 and 16 events, respectively, in All CZP). Overall, 15 RCT CZP patients and 69 All CZP patients were withdrawn from the studies because of SIEs.

### Contribution of age and comorbidities selected for inclusion in age-adjusted comorbidity index

Age ≥ 70 years was strongly associated with the risk of SIEs, contributing 3 points to the AACI score. Diabetes mellitus and COPD/asthma were also important predictors of SIEs and were assigned a weight of 2 points each (Table 1). Other comorbidities contributing to AACI score were hyperlipidemia, osteoporosis, and depression.

Among all patients, the distribution of AACI scores ranged between 0 and 8, and the observed percentage of patients with SIEs generally increased with AACI score (Additional file 1: Table S1). Most patients (80% in both groups) had AACI scores ≤ 1; only one patient in the All CZP group had an AACI score of 8. Owing to the limited number of patients with high AACI scores, those with AACI scores ≥ 2 were pooled in subsequent analyses.

### Identification of baseline risk factors for first SIE

**Fig. 2:**
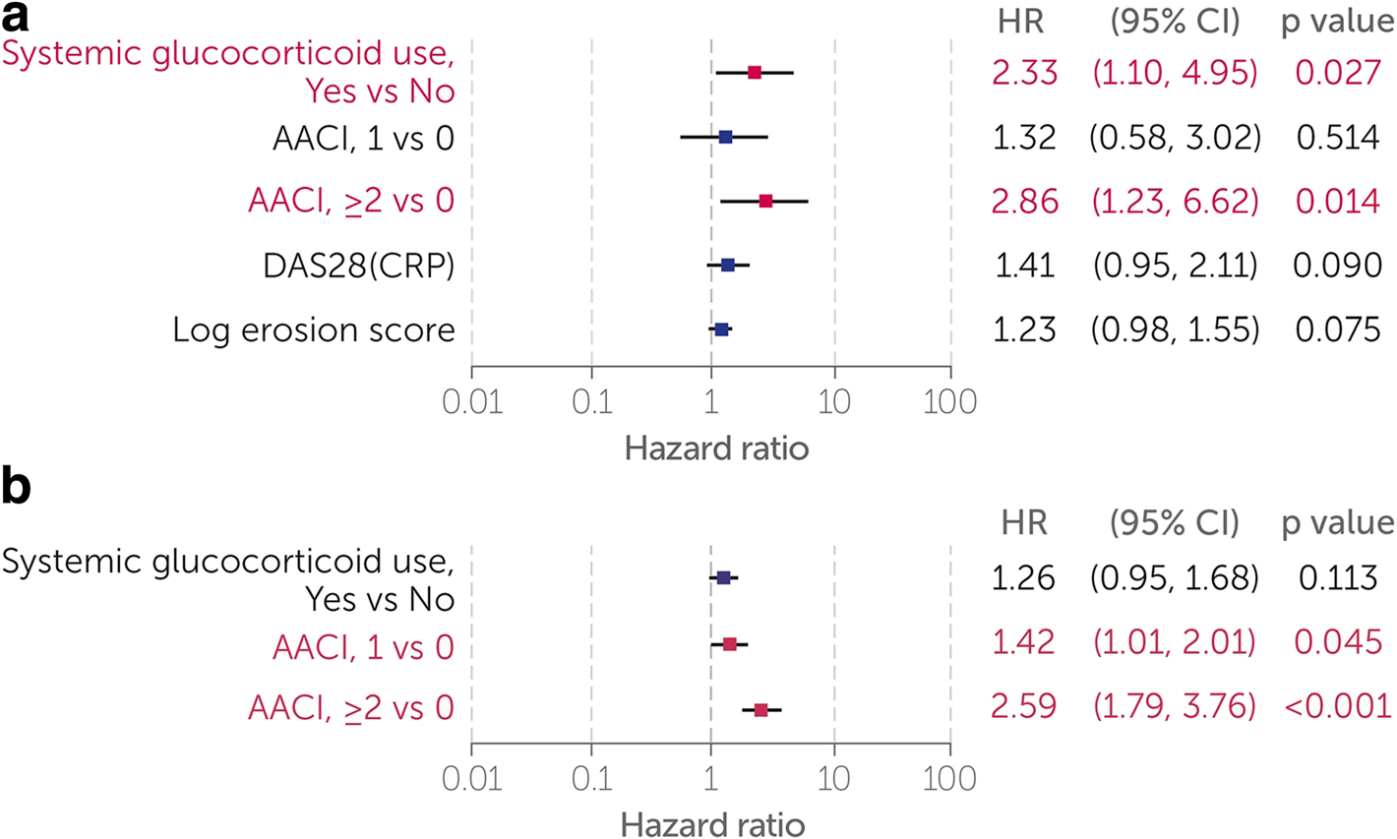
Multivariate analysis of baseline predictors to the first SIE. **a** RCT CZP group. **b** All CZP group. A Cox proportional hazards model fitted with AACI categories was used to identify baseline covariates linked to SIE risk (*p* ≤ 0.25). Baseline covariates identified as risk factors (*p* ≤ 0.05) are highlighted in *red*. No other baseline covariates examined (BMI, disease duration, HAQ-DI, and MTX dose) were considered relevant to the outcome (*p* > 0.25). *SIE* Serious infectious event, *RCT* Randomized controlled trial, *CZP* Certolizumab pegol, *AACI* Age-adjusted comorbidity index, *DAS28(CRP)* 28-joint Disease Activity Score with C-reactive protein, *BMI* Body mass index, *HAQ-DI* Health Assessment Questionnaire Disability Index, *MTX* Methotrexate, *RCT CZP* Patients randomized to CZP in the RAPID1/RAPID2 randomized controlled trials, *All CZP* All patients treated with CZP during the RAPID1/RAPID2 randomized controlled trials and/or open label extensions

AACI scores ≥ 2 and baseline systemic glucocorticoid use were identified by the Cox model as the main risk factors for the first SIE (Fig. 2). In both RCT CZP and All CZP, an AACI ≥ 2 was associated with an increase in the risk of SIEs compared with AACI of 0 (RCT CZP HR 2.86 [1.23–6.62]; All CZP HR 2.59 [1.79–3.76]), and an AACI of 1 was also associated with increased SIE risk in All CZP (HR 1.42 [1.01–2.01]). Baseline systemic glucocorticoid use was also associated with an increased risk of SIEs in RCT CZP patients (HR 2.33 [1.10–4.95]). The association was weaker in the All CZP group (HR 1.26 [0.95–1.68]). In a sensitivity analysis examining different baseline glucocorticoid doses, the risk of SIEs was similar between glucocorticoid doses > 0–5 mg/day and > 5 mg/day (data not shown).

Baseline DAS28(CRP) and erosion scores did not contribute to SIE risk in the All CZP group, but they showed a modest association in the RCT CZP group. No other covariates tested (BMI, disease duration, HAQ-DI, and MTX dose) showed a detectable association with the risk of SIEs (*p* > 0.25). Replacing DAS28(CRP) in the Cox model with alternative measures of disease activity (CRP, DAS28[erythrocyte sedimentation rate], CDAI, and SDAI) produced results similar to those of the main analyses (data not shown).

### Use of baseline age-adjusted comorbidity index and systemic glucocorticoids to predict rate of SIEs

**Fig. 3:**
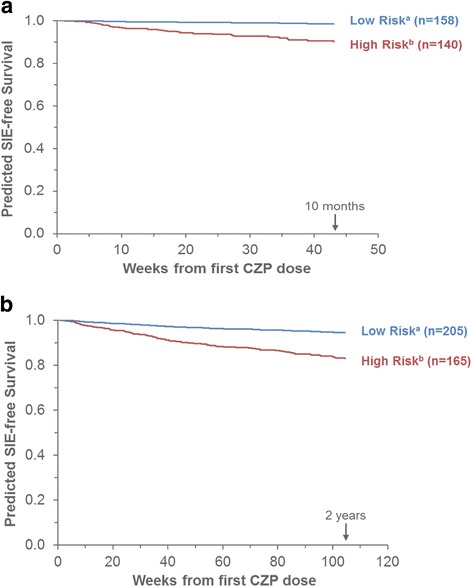
Predicted time to first SIE, by baseline systemic glucocorticoid use and AACI. **a** RCT CZP. **b** All CZP. Predicted SIE-free survival curves correspond to Kaplan-Meier estimates for the indicated risk groups, based on the covariates selected by the Cox models. The c-indexes were 0.66 (95% CI 0.33–0.93) for the RCT CZP model and 0.85 (95% CI 0.73–0.93) for the All CZP model. ^a^Low risk at baseline: AACI of 0, without systemic glucocorticoid use. ^b^High risk at baseline: AACI ≥ 2, with systemic glucocorticoid use. *SIE* Serious infectious event, *CZP* Certolizumab pegol, *AACI* Age-adjusted comorbidity index, *RCT CZP* Patients randomized to CZP in the RAPID1/RAPID2 randomized controlled trials, *All CZP* All patients treated with CZP during the RAPID1/RAPID2 randomized controlled trials and/or open label extensions, *CI* Confidence interval

In the RCT CZP group, the predicted risk of SIEs was lowest for patients with an AACI of 0 and no systemic glucocorticoid use at baseline, and highest in patients with an AACI ≥ 2 who used systemic glucocorticoids (Fig. 3a). Within the same AACI category, the percentage of patients reporting a first SIE was approximately twofold higher if patients also used systemic glucocorticoids at baseline. The c-index (0.66 [0.33–0.93]) indicated fair discrimination between patient groups.

In the All CZP group, the predicted risk of SIEs was lowest for the patient groups defined by AACI of 0 and highest for patients with AACI ≥ 2, regardless of systemic glucocorticoid use (Fig. 3b). The c-index (0.85 [0.73–0.93]) suggested excellent discrimination between patient groups.

Across the different risk groups, observed SIE rates generally matched model predictions, demonstrating that the prediction model was well-calibrated (Table 3). Consistent with model predictions, the observed IRs of SIEs were higher for patients with AACI ≥ 2 and baseline systemic glucocorticoid use, and lower for patients with AACI of 0 and no baseline systemic glucocorticoid use (Table 4).

**Table 3:** Predicted and observed percentages of patients affected by first serious infectious event

Time since first CZP dose	Data						
RCT CZP	Percentage of patients affected by the first SIE^a^	Without systemic glucocorticoid use			With systemic glucocorticoid use		
		AACI = 0 (*n* = 158)	AACI = 1 (*n* = 250)	AACI ≥ 2 (*n* = 110)	AACI = 0 (*n* = 271)	AACI = 1 (*n* = 295)	AACI ≥ 2 (*n* = 140)
3 months	Predicted (95% CI)	0.5 (0.0–1.0)	0.7 (0.1–1.3)	1.4 (0.2–2.7)	1.2 (0.3–2.1)	1.7 (0.6–2.8)	3.8 (1.4–6.1)
	Observed	0.6	1.2	1.8	1.1	2.4	2.9
6 months	Predicted (95% CI)	0.9 (0.1–1.8)	1.2 (0.3–2.2)	2.5 (0.4–4.5)	2.1 (0.6–3.5)	3.0 (1.2–4.7)	6.5 (2.7–10.1)
	Observed	0.6	1.6	2.7	2.2	3.7	5.0
1 year	Predicted (95% CI)^b^	1.5 (0.2–2.8)	1.9 (0.4–3.4)	3.9 (0.7–7.0)	3.3 (1.0–5.5)	4.7 (2.0–7.2)	10.0 (4.3–15.4)
	Observed	0.6	2.0	3.6	3.0	4.1	7.1
All CZP	Percentage of patients affected by the first SIE^a^	Without systemic glucocorticoid use			With systemic glucocorticoid use		
		AACI = 0 (*n* = 205)	AACI = 1 (*n* = 302)	AACI ≥ 2 (*n* = 130)	AACI = 0 (*n* = 325)	AACI = 1 (*n* = 379)	AACI ≥ 2 (*n* = 165)
3 months	Predicted (95% CI)	0.9 (0.4–1.3)	1.2 (0.6–1.8)	2.2 (1.1–3.3)	1.1 (0.6–1.6)	1.5 (0.8–2.2)	2.8 (1.5–4.1)
	Observed	0.5	1.0	1.5	0.9	2.1	3.0
6 months	Predicted (95% CI)	1.6 (0.9–2.3)	2.3 (1.4–3.2)	4.2 (2.5–5.8)	2.1 (1.2–2.9)	2.9 (1.9–3.9)	5.2 (3.3–7.2)
	Observed	2.0	1.3	2.3	2.2	3.7	5.5
1 year	Predicted (95% CI)	3.3 (2.1–4.5)	4.6 (3.2–6.1)	8.3 (5.5–11.0)	4.1 (2.8–5.5)	5.8 (4.1–7.5)	10.4 (7.2–13.4)
	Observed	4.4	2.0	4.6	4.0	7.1	10.9
2 years	Predicted (95% CI)	5.5 (3.6–7.4)	7.7 (5.5–9.9)	13.7 (9.5–17.7)	6.9 (4.8–8.9)	9.6 (7.2–12.0)	16.9 (12.3–21.3)
	Observed	5.9	4.6	13.8	6.8	9.8	12.7

*Abbreviations: SIE* Serious infectious event, *CZP* Certolizumab pegol, *AACI* Age-adjusted comorbidity index, *RCT CZP* Patients randomized to CZP in the RAPID1/RAPID2 randomized controlled trials, *All CZP* All patients treated with CZP during the RAPID1/RAPID2 randomized controlled trials and/or open label extensions, *CI* Confidence interval

^a^Percentage of patients affected by the first SIE up to the indicated time since the first CZP dose

^b^One-year predicted rates in RCT CZP correspond to 301 days (~10 months) because Kaplan-Meier estimates were not defined beyond the last SIE date

**Table 4:** Observed incidence of serious infectious events in patients categorized by age-adjusted comorbidity index and baseline systemic glucocorticoid use

Time since first CZP dose	IR/100 PY (95% CI)^a^					
	Without systemic glucocorticoid use			With systemic glucocorticoid use		
	AACI = 0	AACI = 1	AACI ≥ 2	AACI = 0	AACI = 1	AACI ≥ 2
RCT CZP						
Patients, *n*	158	250	110	271	295	140
3 months	2.6 (0.1–14.5)	4.9 (1.0–14.3)	7.8 (1.0–28.2)	4.5 (0.9–13.3)	9.8 (3.9–20.2)	11.9 (3.2–30.5)
6 months	1.4 (0.0–8.0)	3.7 (1.0–9.6)	6.8 (1.4–19.9)	5.1 (1.9–11.1)	8.6 (4.3–15.4)	12.1 (4.9–25.0)
1 year	1.0 (0.0–5.3)	3.2 (1.0–7.4)	6.2 (1.7–15.9)	4.6 (2.0–9.1)	6.2 (3.2–10.9)	12.1 (5.8–22.2)
All CZP						
Patients, *n*	205	302	130	325	379	165
3 months	2.0 (0.1–11.1)	4.1 (0.8–11.9)	6.6 (0.8–23.7)	3.8 (0.8–11.1)	8.7 (3.8–17.1)	12.6 (4.1–29.4)
6 months	4.1 (1.1–10.5)	2.8 (0.8–7.1)	5.1 (1.0–14.8)	4.5 (1.8–9.3)	7.8 (4.3–13.1)	11.7 (5.4–22.2)
1 year	4.8 (2.2–9.1)	2.2 (0.8–4.7)	5.3 (2.0–11.6)	4.3 (2.3–7.4)	8.0 (5.3–11.7)	12.6 (7.5–20.0)
2 years	3.4 (1.8–6.0)	2.7 (1.5–4.5)	8.9 (5.3–14.0)	3.9 (2.5–5.9)	6.0 (4.3–8.3)	8.4 (5.2–12.8)

*Abbreviations: SIE* Serious infectious event, *CZP* Certolizumab pegol, *IR* Incidence rate, *PY* Patient-years, *AACI* Age-adjusted comorbidity index, *RCT CZP* Patients randomized to CZP in the RAPID1/RAPID2 randomized controlled trials, *All CZP* All patients treated with CZP during the RAPID1/RAPID2 randomized controlled trials and/or open label extensions, *CI* Confidence interval

^a^IR of SIEs observed during the indicated time interval since the first day on CZP

As a sensitivity analysis, the RABBIT Risk Score was tested in the All CZP group. On the basis of expected SIE risk, patients were assigned only to the first three deciles of RABBIT Risk Scores published by Zink et al*.* [17]. Predicted SIE rates were generally comparable to observed rates, with some underestimation of SIE rates in patients with lower RABBIT Risk Scores (Additional file 1: Figure S1).

### Observed rates of LDA and remission in different SIE risk groups

**Fig. 4:**
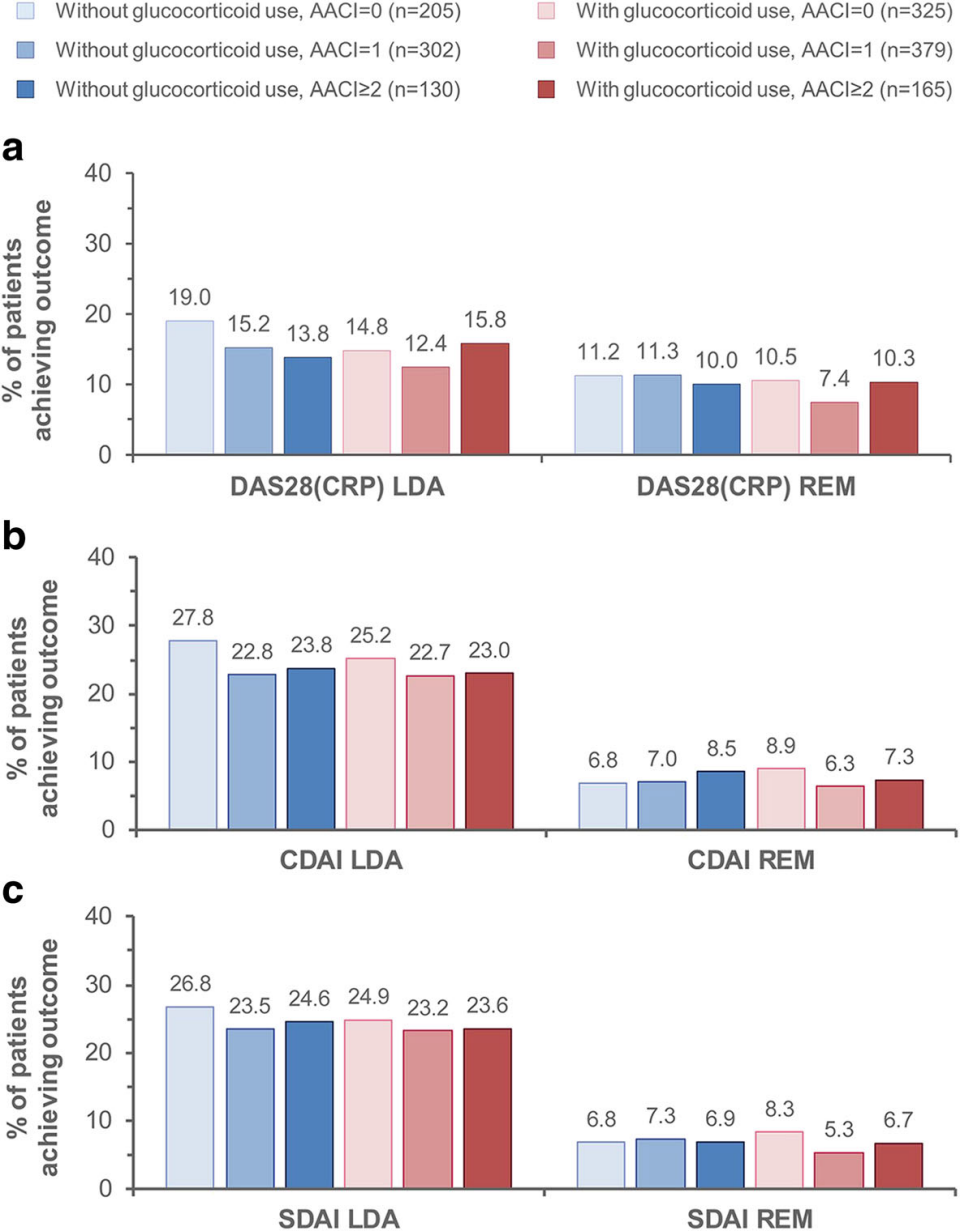
Observed proportion of All CZP patients achieving LDA and remission at 1 year of treatment. **a** DAS28(CRP). **b** CDAI. **c** SDAI. LDA was defined as DAS28(CRP) ≤ 2.7, CDAI ≤ 10, and SDAI ≤ 11; remission corresponded to DAS28(CRP) < 2.3, CDAI ≤ 2.8, and SDAI ≤ 3.3. Missing data were imputed using nonresponder imputation. *LDA* Low disease activity, *CZP* Certolizumab pegol, *AACI* Age-adjusted comorbidity index, *DAS28(CRP)* 28-joint Disease Activity Score with C-reactive protein, *REM* Remission, *CDAI* Clinical Disease Activity Index, *SDAI* Simplified Disease Activity Index, *All CZP* All patients treated with CZP during the RAPID1/RAPID2 randomized controlled trials and/or open label extensions

We investigated if the predicted SIE risk influenced the achievement of LDA and remission at 1 year of CZP treatment according to DAS28(CRP), CDAI, and SDAI criteria (Fig. 4). In All CZP, the percentage of patients achieving LDA was comparable across the different SIE risk groups, albeit with a numerically higher proportion in the low-risk group (AACI of 0 and no baseline systemic glucocorticoid use). Likewise, the rates of remission did not vary considerably between SIE risk groups.

## Discussion

Patients with RA are reported to have an increased susceptibility to infection compared with patients without RA [28]. Although the strength of association between anti-TNFs and the risk of infection remains a topic of debate [2–5], the evidence published so far underscores the need for clinicians to carefully balance the clinical benefits and potential harms of initiating anti-TNF therapy in individual patients.

Using data pooled from the pivotal RAPID1 and RAPID2 RCTs and OLEs [20–23], we derived and tested a new AACI to predict the risk of SIEs during CZP treatment, based on the baseline characteristics of anti-TNF naive patients with moderate to severe active RA and inadequate response to prior disease-modifying antirheumatic drugs. AACI ≥ 2 and systemic glucocorticoid use were identified as the main baseline risk factors for the occurrence of SIEs. With up to 2 years of CZP exposure, predicted SIE rates matched the observed rates, suggesting that the model was well-calibrated for the outcome. Discrimination between different risk groups in the model was fair (RCT CZP) to excellent (All CZP).

As expected, the IRs of SIEs in groups defined as being at high risk were higher than reported previously in a pooled safety analysis of patients with RA in CZP clinical trials (IR 3.7/100 PY [3.3–4.1] [25]). Consistent with previous CZP safety data [25] and registry data [10, 13], the highest incidence of SIEs was seen during the first 6 months of anti-TNF treatment and decreased thereafter.

In All CZP, the rates of LDA and remission observed at 1 year were similar across all six risk groups, suggesting that the baseline risk of SIEs was not associated with the likelihood of response to CZP treatment. Indeed, poor prognostic factors for RA progression, such as longer disease duration, greater disability, and erosive disease [29, 30], were not associated with SIE risk when tested in the Cox model.

In line with previous studies [9–14], older age (≥70 years), diabetes mellitus, and COPD/asthma were strongly associated with SIE risk in this RA population, making an important contribution to patients’ AACI scores. Consistent with our results, older age and comorbidities also featured in other SIE risk scores in RA, developed using data from biologics registries and administrative databases [11, 16, 17]. In addition, we found that hyperlipidemia, osteoporosis, and depression were associated with an increased risk of SIEs in the RAPID1/RAPID2 patient population. Depression and the use of certain antidepressant medications have previously been associated with an increased risk of some infections [31]. However, it is uncertain whether some of these associations might have a true biologic basis, be driven by the effects of medications used to treat the condition, be a proxy for a shared risk factor (e.g., frailty), or reflect other confounding factors.

The increased risk of infection associated with systemic glucocorticoids is widely recognized [6, 8, 12, 15]. Our data revealed a dose-independent association between baseline systemic glucocorticoid use and SIEs in the RCT CZP group, where doses were required to remain stable throughout the RCTs [20, 21]. By contrast, systemic glucocorticoid doses were allowed to change during the OLEs, which may have minimized our ability to detect a stronger association with SIE risk in the All CZP group. Furthermore, we cannot discount the possibility that doses taken over the previous 2–3 years may have had a cumulative impact on the risk of SIEs [32]. Despite such limitations, our results suggest potential benefits of avoiding concomitant systemic glucocorticoid use in CZP-treated patients with RA as early as possible, as recommended by the 2015 American College of Rheumatology RA guidelines [33].

High disease activity has been associated with an increased risk of infection [34, 35]. However, in our model, baseline disease activity showed only a modest association with the risk of SIEs. One likely explanation is the fact that all patients enrolled in the RAPID1/RAPID2 RCTs had moderate to high disease activity at baseline. The relatively narrow range of baseline DAS28(CRP) scores in this RA population may have concealed a stronger association with SIE risk.

Several studies have reported that the relative risk of infection decreases over time in patients with RA treated with anti-TNF [13, 25, 36–38]. This has been attributed not only to the depletion of susceptible patients from the study cohorts but also to an improvement in the clinical status of patients, either as a direct result of reduced disease activity or owing to a decline in the use of concomitant glucocorticoids [8]. The model that we developed tested only the effect of baseline patient characteristics. Although AACI, which is a static patient characteristic, was the strongest predictor of SIEs, clinical parameters such as disease activity and physical function (HAQ-DI) may have changed considerably over time as a result of CZP treatment, resulting in weaker prediction of SIE rates for CZP exposures > 2 years.

A limitation of the method used to derive the AACI is the fact that the HRs for each age category and individual comorbidity were averaged across the RCT CZP and All CZP groups in order to assign the respective weighting. Our intention with the AACI was to provide an indication of SIE risk not only in the first year of treatment but also over a longer period of time. Had we used just the RCT period to derive the weightings for each age category and comorbidity, we would have potentially overestimated the risk of SIEs during the OLE. For this reason, the averaging of HRs seemed to be a reasonable approach, albeit at the expense of some predictive accuracy.

Prior epidemiologic work has suggested that although some risk estimation models can be generalized, model performance is optimized when population-specific weights can be derived [11]. As such, the weighting for each risk factor in one population may not be generalizable to a different RA population. For instance, the RABBIT Risk Score performed well in the All CZP group, but SIE rates were underestimated at the lower end of the risk spectrum. One possible explanation is the fact that the RABBIT Risk Score may be better calibrated for real-world patient populations with a higher propensity for SIEs [17]. For example, chronic renal disease and chronic lung disease, which contribute to the RABBIT Risk Score, were less prevalent or absent in our study population. Furthermore, our assumption that there were zero patients with a history of prior SIEs may also have contributed to the underestimation of RABBIT Risk Scores.

At initiation of anti-TNF therapy, prescribers need to consider the infection risk in each individual patient, which must take into account the patient’s age, comorbidities, concomitant medications, and clinical history. However, most data currently available consist of mean infection rates observed in relatively large, heterogeneous populations. Although group-level infection rates are informative, they are not always relevant at the individual patient level in clinical practice. This is why stratifying patients according to comorbidity burden and other infection risk factors can help clinicians to make more accurate risk-benefit assessments regarding anti-TNF therapy and ultimately make better-informed treatment decisions at the individual-patient level.

However, in clinical practice, prescribers tend to channel higher-risk patients away from anti-TNF therapy, owing to the commonly held view that older patients or those with more severe comorbidities are less likely to benefit clinically from anti-TNF treatment [11, 39, 40]. However, there is published evidence suggesting that responsiveness to treatment and the incremental infection risk associated with anti-TNFs is similar for higher- and lower-risk groups [41, 42]. Consistent with this, our data showed that the rates of LDA and remission at 1 year of CZP treatment were similar across all AACI risk groups. Although the limited placebo exposure did not allow us to quantify the incremental risk associated with CZP, the results of our study are reassuring for patients with higher AACI scores at baseline and may encourage clinicians to avoid concomitant glucocorticoids to reduce the SIE risk. Further research is needed to examine how the disease-modifying effects of CZP treatment, as well as changes in glucocorticoid use, influence the risk of SIEs in a time-dependent manner.

## Conclusions

In this population of patients with moderate to severe active RA and no prior anti-TNF use, the rate of SIEs during CZP treatment was predicted with considerable accuracy using a combination of AACI score and baseline systemic glucocorticoid use, with good discrimination between high- and low-risk patient groups. This SIE risk score may provide a valuable tool for clinicians when considering the risk of infection in individual patients with RA. One of the greatest strengths of our analysis was the use of a large, very comprehensive clinical trial database to derive the AACI. However, we should note that the AACI model has not been validated in a different population. It would be valuable to assess how the AACI performs in other RA trials and in real-world settings.

## Additional files

Additional file 1: Methods: testing of RABBIT Risk Score in the All CZP group. **Table S1.** Observed number of SIEs in patients categorized by AACI. **Figure S1.** Predicted and observed SIE rates in All CZP patients according to the RABBIT Risk Score. (PDF 178 kb)

## Abbreviations

AACI: Age-adjusted comorbidity index
BMI: Body mass index
CDAI: Clinical Disease Activity Index
CI: Confidence interval
COPD: Chronic obstructive pulmonary disease
CRP: C-reactive protein
CZP: Certolizumab pegol
DAS28(CRP): 28-joint Disease Activity Score with C-reactive protein
HAQ-DI: Health Assessment Questionnaire Disability Index
HR: Hazard ratio
IR: Incidence rate
LD: Loading dose
LDA: Low disease activity
MedDRA: Medical Dictionary for Regulatory Activities
mTSS: Modified total Sharp score
MTX: Methotrexate
OLE: Open-label extension
PY: Patient-years
RA: Rheumatoid arthritis
RABBIT: Rheumatoid Arthritis Observation of Biologic Therapy (the German biologics register)
RAPID: Rheumatoid Arthritis PreventIon of structural Damage
RCT: Randomized controlled trial
SAE: Serious adverse event
SDAI: Simplified Disease Activity Index
SIE: Serious infectious event
TNF: Tumor necrosis factor
