# Supplementary material for: Use of a baseline risk score to identify the risk of serious infectious events in patients with rheumatoid arthritis during certolizumab pegol treatment
Source: Arthritis Res Ther. 2017 Dec 15;19:276. doi: 10.1186/s13075-017-1466-y (PMC5732488; doi:10.1186/s13075-017-1466-y)
Supplement: Supplementary file 1 — Methods: testing of RABBIT Risk Score in the All CZP group. Table S1. Observed number of SIEs in patients categorized by AACI. Figure S1. Predicted and observed SIE rates in All CZP patients according to the RABBIT Risk Score. (PDF 178 kb) [file 13075_2017_1466_MOESM1_ESM.pdf]

# Use of a Baseline Risk Score to Identify the Risk of Serious Infectious Events in Patients with Rheumatoid Arthritis During Certolizumab Pegol Treatment

Jeffrey R. Curtis, Kevin Winthrop, Cathy O'Brien, 'Matladi N. Ndlovu, Marc de Longueville, Boulos Haraoui

## ADDITIONAL FILE 1

## ADDITIONAL METHODS

### Testing of RABBIT risk score in the All CZP group

As a sensitivity analysis, the German biologics register (RABBIT) risk score[1] was tested in the All CZP group. Predicted rates of SIEs were based on patients' RABBIT risk scores at baseline, adjusted for duration of CZP exposure. Patients were then stratified into three subgroups, according to the expected SIE risk established by the RABBIT risk score; these three subgroups were similar to the first three deciles of expected risk published in Zink *et al.*[1] The SIE rates predicted by the RABBIT risk score were compared with observed SIE rates.

The risk factors included in the calculation of RABBIT risk scores were age >60 years (Yes; No), HAQ-DI, chronic lung disease (Yes; No), mean glucocorticoid dose (7.5–14 mg/day; ≥15 mg/day), and anti-TNF treatment ("Yes" for all patients). The remaining risk factors which feature in the RABBIT risk score were set to zero, either because no patients included in the study population presented those characteristics (chronic renal disease, >5 previous treatment failures), or due to insufficient information (history of prior SIEs). Patients with a history of chronic infection, recent serious or life-threatening infection within 6 months from screening, or with signs or symptoms that may have indicated an infection, were excluded from participating in RAPID1/RAPID2, as were patients with current or recent history of severe, progressive, and/or uncontrolled renal disease.[2, 3]

**Additional Table S1.** Observed number of SIEs in patients categorized by AACI

| | **RCT CZP (n=1224; 798.5 PY)** | | **All CZP (n=1506; 5778.6 PY)** | |
|---|---|---|---|---|
| | Patients, n | SIEs (n, %)[a] | Patients, n | SIEs (n, %)[a] |
| **AACI=0** | 429 | 9 (9, 2%) | 530 | 61 (52, 10%) |
| **AACI=1** | 545 | 17 (17, 3%) | 681 | 100 (88, 13%) |
| **AACI=2** | 39 | 2 (2, 5%) | 45 | 11 (8, 18%) |
| **AACI=3** | 150 | 6 (5, 3%) | 179 | 45 (37, 21%) |
| **AACI=4** | 13 | 0 | 15 | 8 (5, 33%) |
| **AACI=5** | 36 | 4 (4, 11%) | 41 | 6 (6, 15%) |
| **AACI=6** | 8 | 2 (2, 25%) | 8 | 2 (2, 25%) |
| **AACI=7** | 4 | 1 (1, 25%) | 6 | 3 (3, 50%) |
| **AACI=8** | 0 | 0 | 1 | 0 |
| **AACI≥2** | 250 | 15 (14, 6%) | 295 | 75 (61, 21%) |

[a]Results are shown as total number of observed SIEs (number of patients affected by SIEs, corresponding percentage among patients with the same AACI score). SIE: serious infectious event; RCT: randomized controlled trial; CZP: certolizumab pegol; PY: patient-years; AACI: age-adjusted comorbidity index

**Additional Figure S1.** Predicted and observed SIE rates in All CZP patients according to the RABBIT risk score

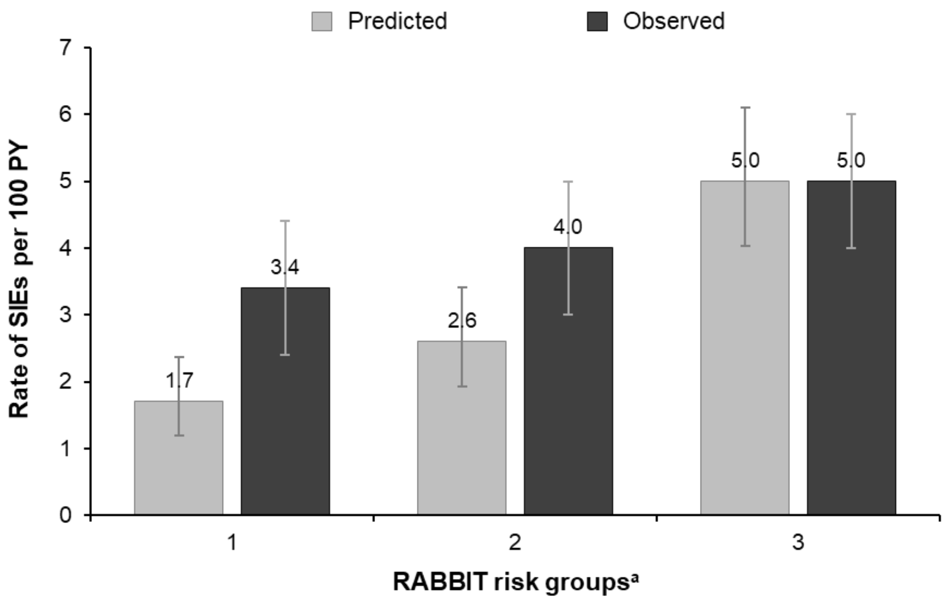


Predicted rates of SIEs were based on patients' RABBIT risk scores, adjusted for duration of CZP exposure, and were compared to the observed SIE rates; vertical bars correspond to 95% confidence intervals. [a]Patients were stratified into three subgroups, according to the expected SIE risk established by the RABBIT risk score; these three subgroups were similar to the first three deciles of expected risk published by Zink *et al.*[1] SIE: serious infectious event; CZP: certolizumab pegol; RABBIT: Rheumatoide Arthritis Beobachtung der Biologika-Therapie (Rheumatoid Arthritis Observation of Biologic Therapy, the German biologics registry); PY: patient-years.

## REFERENCES

1. Zink A, Manger B, Kaufmann J, Eisterhues C, Krause A, Listing J, Strangfeld A: **Evaluation of the RABBIT Risk Score for serious infections**. *Annals of the rheumatic diseases* 2014, **73**(9):1673-1676.

2. Keystone E, Heijde D, Mason D, Jr., Landewe R, Vollenhoven RV, Combe B, Emery P, Strand V, Mease P, Desai C *et al*: **Certolizumab pegol plus methotrexate is significantly more effective than placebo plus methotrexate in active rheumatoid arthritis: findings of a fifty-two-week, phase III, multicenter, randomized, double-blind, placebo-controlled, parallel-group study**. *Arthritis and rheumatism* 2008, **58**(11):3319-3329.

3. Smolen J, Landewe RB, Mease P, Brzezicki J, Mason D, Luijtens K, van Vollenhoven RF, Kavanaugh A, Schiff M, Burmester GR *et al*: **Efficacy and safety of certolizumab pegol plus methotrexate in active rheumatoid arthritis: the RAPID 2 study. A randomised controlled trial**. *Annals of the rheumatic diseases* 2009, **68**(6):797-804.
